# Comparison of core muscle strengthening exercise and stretching exercise in middle-aged women with fibromyalgia: A randomized, single-blind, controlled study

**DOI:** 10.1097/MD.0000000000027854

**Published:** 2021-12-17

**Authors:** Hyeng-Kyu Park, Min-Keun Song, Dong-Joo Kim, In-Sung Choi, Jae-Young Han

**Affiliations:** a Department of Physical & Rehabilitation Medicine, Chonnam National University Bitgoeul hospital, Gwangju, Republic of Korea; b Department of Physical & Rehabilitation Medicine, Regional Cardiocerebrovascular Center, Center for Aging and Geriatrics, Chonnam National University Medical School and Hospital, Gwangju, Republic of Korea.

**Keywords:** balance, fibromyalgia, pain, strengthening, stretching

## Abstract

**Background:**

Many studies have reported that exercise is effective for fibromyalgia and various types of exercise are recommended. However, most of exercises lack evidence for fibromyalgia symptoms. We aimed to examine the effect of core muscle strengthening exercise compared to general stretching exercise in fibromyalgia patients.

**Methods:**

Forty fibromyalgia patients were enrolled. They were provided exercise program twice a week for 4 weeks: core muscle strengthening exercise and general stretching exercise.

Outcome measures were Visual Analogue Scale, Borg Scale, fibromyalgia impact questionnaire (FIQ), widespread pain index, Symptom Severity Scale (SS), and balance scale and measured before and after exercise program. Balance function was assessed by checking the distance of sway on soft pad with eyes open (EO) and with eyes closed (EC).

**Results:**

After program, FIQ, SS, EO, and eyes closed showed statistically significant differences in the strengthening group while Visual Analogue Scale, Borg scale, FIQ, widespread pain index, SS showed statistically significant differences in stretching group. And EO showed statistically significant differences in the intergroup analysis.

**Conclusions:**

Both exercise could improve symptoms of fibromyalgia but showed no significantly better efficiency with intergroup analysis. Only some balance function was improved with core muscle strengthening exercise with significant difference. Our study presents preliminary results regarding the comparison between both exercises for fibromyalgia through a randomized controlled trial.

## 1 Introduction

Fibromyalgia is a medically unexplained syndrome characterized by chronic widespread pain, fatigue, reduced physical function, tender points on the body, and sleep disorders.^[1–3]^ It may reduce the quality of life of patients by affecting their daily activities.^[1]^ The prevalence of fibromyalgia within the general population appears to range from 1.3% to 8%.^[4]^

Many systematic reviews and articles have explained about the diagnosis, mechanism, and treatment of fibromyalgia.^[5,6]^ However, the exact mechanism of fibromyalgia still remains unclear. Some articles reported about the role of central sensitization as the main pathogenetic process in fibromyalgia.^[7,8]^ On the other hand, other article reported a more peripheral abnormality with small fiber neuropathy.^[9]^

Symptoms of fibromyalgia vary among patients. Most patients suffer from severe pain. Besides pain, reduced postural stability and increased frequency of falls are also frequently found in fibromyalgia patients.^[10–12]^ For the diagnosis, there are various criteria including history of pain, family history, somatic symptoms and others.^[5]^

For its treatment, combination of pharmacological and nonpharmacological treatment is required.^[2,13]^ However, pharmacological treatments are not good enough for managing symptoms of fibromyalgia. For nonpharmacological treatment, many types of exercise are commonly recommended.^[14,15]^ However, further study is required.

Generally, stretching exercise is prescribed for the treatment of fibromyalgia.^[6,15]^ European League Against Rheumatism has reported that exercise including aerobic exercise and strengthening exercise is efficient for pain.^[6]^ Other studies have reported the effect of many types of exercise for fibromyalgia.^[15–18]^ Through the studies, exercise is strongly recommended regardless of type of exercise.^[6]^ However, there is few studies that compared the efficacy of each exercise. Further, there is no study that compared core muscle strengthening exercise and general stretching exercise.

Besides pain management, increased falling risk and postural instability in fibromyalgia patients also need evaluation and management.^[12]^ In general, core muscle exercise is effective for low back pain and postural stability. Considering the frequency of fall and postural instability in fibromyalgia patients, core muscle stability is important for them. Some studies have reported that impaired postural control and low balance are associated with pain and symptom severity and reported that core strengthening exercise is more efficient than traditional pilates training.^[10,19]^ However, no study has compared core muscle strengthening exercise and general stretching exercise for patients with fibromyalgia. Therefore, we aimed to compare the effect of core muscle strengthening exercise with general stretching exercise on symptoms including balance in fibromyalgia patients.

## 2 Methods

### 2.1 Study design – randomization and masking

This study is a prospective, single-blinded (outcome assessor), parallel-group, randomized controlled clinical trial with a 1:1 ratio of participants to be allocated into the core muscle strengthening exercise group or general stretching exercise groups. In this study, each participant will know which program is applied to them. Therefore, we have adopted a single blinding (outcome assessor blinding) approach. To maintain the objectivity of this study, we have separated the outcome assessor, clinical research coordinator, and therapist who treats the participant. In this study, individuals with conflicts of interest or preconceived positions are excluded to prevent related bias. For the randomization, we used randomization allocation software program (M. Saghaei, MD., Isfahan Medical Science, Isfahan, Iran) and participants were randomly allocated 20 participants into each group. This randomization process will be conducted by a third-party team of statisticians and the allocations will be concealed and sequentially numbered with opaque and sealed envelopes. The serial number codes will be opened in the presence of the participants and treatment team without assessor. And only the principle investigator will generate the allocation sequence, enrollment, and assign participants to interventions.

This study was approved by the Chonnam National University Hospital Institutional Review Board (CNUH-2016–183). It was registered in the Clinical Research Information Service (CRIS: KCT0003111).

### 2.2 Participants

We screened fibromyalgia patients diagnosed by American College of Rheumatology preliminary diagnostic criteria 2010 who visited our Rheumatology and Rehabilitation center.^[3]^

Inclusion criteria were: those who could walk independently, aged from 35 to 65 years, no change of medication for 3 months, no events that could influence walking ability, and no surgical history for previous 6 months. Exclusion criteria were: pregnant women, patients with acute disease including high fever and uncontrolled medical conditions (such as severe infection and cardiovascular disease including unstable angina), and who could not perform exercise program.

**Figure 1:**
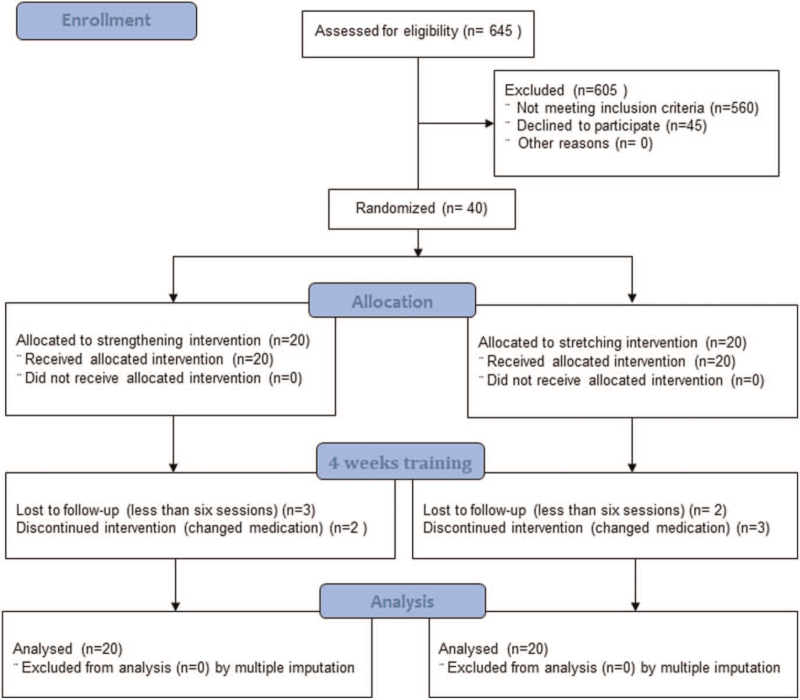
Participants flow diagram (the CONSORT flow diagram).

Patients were randomly allocated into core muscle strengthening exercise group (20 patients, strengthening group) and general stretching exercise group (20 patients, stretching group). The randomization process was performed by a person who was not involved in any other study procedure. All participants were informed of the consent of this study. Patient recruitment, allocation, and retention are shown in Figure 1.

### 2.3 Interventions

Each participant performed exercise sessions twice a week of 30 minutes in duration for 4 weeks at our rehabilitation center. Evaluation of participants was performed by 1 doctor and 1 physical therapist at the first and the final visit. The investigator who performed pre- and post-treatment outcome measurements remained blind to group allocation. Exercise sessions were performed by the same physical therapist who were not engaged in evaluation.

**Figure 2:**
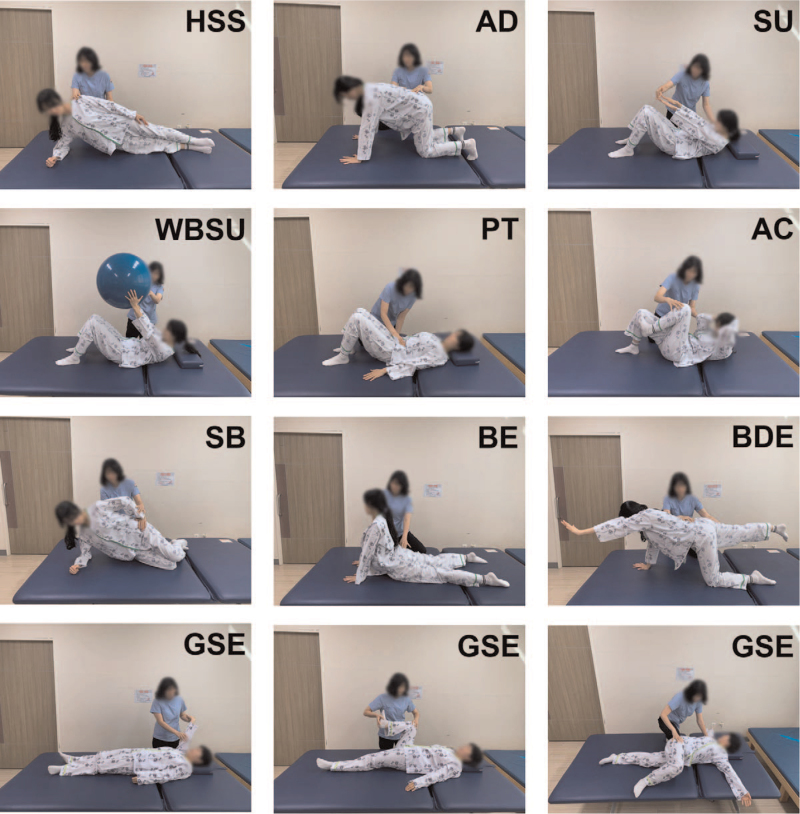
Images of the core muscle strengthening exercise (HSS, AD, SU, WBSU, PT, AC, SB, BE, BDE) and GSE. AC = abdominal crunches, AD = abdominal drawing in maneuver, BDE = bird dog exercise, BE = back extension, GSE = general stretching exercise, HSS = horizontal side support, PT = pelvic tilting, SB = side bridge, SU = sit up, WBSU = weight bearing sit up.

During each session, light dynamic stretching was performed for 5 minutes in both groups before and after main exercise (20 minutes). Core muscle strengthening exercise was programmed with horizontal side support, abdominal drawing in maneuver, sit up, weight bearing sit up, pelvic tilting, abdominal crunches, side bridge, back extension, and bird dog exercise.^[20–24]^ General stretching exercise was focused on main areas of pain to minimize tender point location and individualized to teach each participant to locate their stop point and avoid overstretching in participants.^[16,17]^ Images of each exercise are shown at Figure 2. In the stretching group, the tender point was determined by the appeal of participant during each visit and exercise was composed of 2 sets of 3 repetitions for 8 to 9 different exercises maintaining the stretched position for 30 seconds. Stretching exercise was mainly focused on the tender point corresponding to 19 body areas as widespread pain index (WPI).

### 2.4 Outcome measures

All outcome measures were checked at their first and final visits.

Pain was assessed using a Visual Analogue Scale (VAS) within the last week. The current health status of each participant was evaluated with fibromyalgia impact questionnaire (FIQ) including 10 items regarding physical functioning, status of feeling well, inability to go for work, difficulty in working, pain, fatigue, morning fatigue, stiffness, anxiety, and depression. Participants were also asked to indicate in which of 19 body areas they had pain during the last week. The pain was described as WPI. Somatic symptoms, walking unrefreshed, cognition, fatigue, and others during last week were evaluated with Symptom Severity Scale (SS).^[3,5]^ For monitoring and quantifying participant's perceptions of effort during exercise, Borg scale was checked.^[25]^

Balance function was evaluated using I Balance S (Cybermedic Co., Iksan, Korea). Measurements were taken while participant was standing firmly on both feet on the force plate. Images on the computer monitor that is configured to move the dots according to the movement of the center of the body was placed just in front of the platform. The participant was asked to keep balance on soft pad with eyes open (EO) and on soft pad with eyes closed (EC). Balance function was assessed by checking the total sway of the center of pressure.

During 8 sessions of our protocol, we excluded participants who performed <6 sessions from analysis.

### 2.5 Safety assessments

During the study, all unexpected and unintended signs, symptoms, or diseases referred to adverse effects (AEs). During this period, all AEs reported by participants or the investigator were to be recorded in detail, such as when the AEs occur, duration of symptoms, degree of severity (mild, moderate, or severe), any measures related to the AEs, time of AEs disappearance, and any potentially causal relationship between the treatment and AEs. All AEs were to be reported to the principal investigator and IRB and to be documented.

The possible AEs of this protocol included pallor, nausea, vomiting, fracture, local hematoma, sweating or dizziness, fainting, infection, pain, anxiety, and headache.

### 2.6 Sample size and statistical analysis

The primary outcome measure for this study was the change of the mean difference and SD in VAS score and we calculated the sample size with the mean difference and standard deviation like other study that VAS was primary outcome. So, we calculated the sample size of 15 participants per group would be required to provide 80% power at an alpha level of 0.05. Considering drop rate of 25%, the number of sample size was calculated to be 40.^[16,17,26]^

Data are expressed as means ± standard deviation for most variables. Descriptive statistics were calculated and compared for statistical significance. Baseline and after treatment data were compared between the 2 groups using Wilcoxon signed rank test. Changes of variables after each exercise program in both groups were calculated using Mann–Whitney test and effect sizes were calculated. About the missing data, we used multiple imputation methods. *P* values < .05 were considered statistically significant. All statistical analyses were performed using SPSS for Windows ver.23.0 (SPSS Inc., Chicago, IL).

## 3 Results

A total of 645 patients were screened from March 1, 2016 to May 1, 2017. Among them, 560 patients failed to meet the inclusion criteria; 422 patients changed their medication due to pain severity, 108 patients were not according to our inclusion criteria for age, and 30 patients cannot walk or other lesions. After excluding them, we explained our study to eligible patients and 45 patients declined to participate. Finally, 40 fibromyalgia patients were recruited. Among them, 2 men and 38 women participants were assigned for their study protocol. Out of them, we excluded the participants who performed <6 sessions regardless of outcome measurement. In the strengthening group, 3 participants performed <6 sessions due to personal reason and 2 participants changed their medication. In the stretching group, 2 participants performed <6 sessions and 3 participants changed their medication. A total of 5 women performed <6 sessions while 2 men and 3 women participants changed their medication. Totally, 10 participants were excluded. And consequently, 30 women participants completed interventions.

### 3.1 Patient demographics

During study period, no AEs related to our study was observed in either group. Subjects’ baseline demographics and clinical characteristics are shown by mean standard deviation in Table 1. Both strengthening and stretching groups were composed of 20 participants. The 2 groups were well matched at baseline assessment. There was no statistically significant intergroup differences for any parameter analyzed.

**Table 1:** General characteristics of subjects.

	Strengthening group	Stretching group	*P*-value
Age (yr)	52.8 ± 7.1	50.5 ± 7.1	.233
Gender (n)			1.000
Men	1	1	
Women	19	19	
Height (cm)	158.5 ± 5.2	158.3 ± 5.8	.903
Weight (kg)	59.0 ± 8.4	60.3 ± 6.8	.303
BMI (kg/m^2^)	23.5 ± 3.5	24.1 ± 3.1	.449

Values are presented as mean ± standard deviation. Strengthening group: core muscle strengthening exercise group, stretching group: general stretching exercise group. BMI = body mass index.

### 3.2 Symptoms of fibromyalgia

As shown in Tables 2 and 3, FIQ and SS scores were significantly lower in both groups after 4 weeks of exercise program compared to initial assessment. VAS, Borg scale, and WPI scores were significantly lower only in the stretching group after 4 weeks of exercise program compared to initial assessment.

**Table 2:** Therapeutic effects of core muscle strengthening exercise (strengthening group).

	Strengthening group		
Variable	Before exercise	After exercise	*P*-value
VAS	62.5 ± 17.4	54.5 ± 25.2	.083
Borg scale	12.1 ± 1.7	11.8 ± 1.7	.697
FIQ	58.8 ± 20.6	45.5 ± 24.3	.004^∗^
WPI	9.9 ± 4.5	11.1 ± 6.0	.640
SS	7.4 ± 2.4	5.9 ± 2.9	.018^∗^
Balance EO (mm)	310.1 ± 83.9	259.6 ± 55.4	.011^∗^
Balance EC (mm)	457.0 ± 120.6	381.8 ± 67.7	.040^∗^

Values are presented as mean ± standard deviation. EC = sway distance on soft pad with eyes closed, EO = sway distance on soft pad with eyes open, FIQ = fibromyalgia impact questionnaire, SS = Symptom Severity Scale, VAS = Visual Analogue Scale, WPI = widespread pain index.

∗ Statistically significant (*P* < .05) difference between before and after exercise.

**Table 3:** Therapeutic effects of general stretching exercise (stretching group).

	Stretching group		
Variable	Before exercise	After exercise	*P*-value
VAS	68.0 ± 15.4	54.0 ± 13.9	.003^∗^
Borg scale	12.1 ± 2.0	11.0 ± 1.2	.011^∗^
FIQ	66.7 ± 12.7	56.8 ± 16.5	.033^∗^
WPI	9.8 ± 3.4	7.1 ± 3.3	.004^∗^
SS	8.3 ± 1.7	5.8 ± 2.0	.003^∗^
Balance EO (mm)	326.3 ± 68.6	361.6 ± 106.0	.167
Balance EC (mm)	498.0 ± 240.3	468.1 ± 179.1	.695

EC = sway distance on soft pad with eyes closed, EO = sway distance on soft pad with eyes open, FIQ = fibromyalgia impact questionnaire, SS = Symptom Severity Scale, VAS = Visual Analogue Scale, WPI = widespread pain index.

∗ Statistically significant (*P* < .05) difference between before and after exercise.

About the balance, as shown in Tables 2 and 3, EO and EC scores were significantly lower in the strengthening group after 4 weeks of exercise program compared to initial assessment. Changes of variables after exercise program are presented as delta value (Δ value) as shown in Table 4. Δ values of EO and EC were positive in the strengthening group but negative in the stretching group and EO was significantly different within the intergroup analysis.

**Table 4:** Changes of variables after each exercise program in core muscle strengthening exercise (strengthening group) and general stretching exercise group (stretching group).

	Strengthening group	Stretching group	*P*-value	Effect size (95%CI)
ΔVAS	12.62	17.85	.744	0.15 (−0.48∼0.77)
ΔBorg scale	1.31	7.80	.094	0.39 (−0.24∼1.01)
ΔFIQ	22.61	12.74	.185	0.02 (−0.60∼0.64)
ΔWPI	−31.73	24.43	.063	0.75 (0.11∼1.39)
ΔSS	17.21	26.16	.473	0.40 (−0.22∼1.03)
ΔBalance EO (mm)	11.87	−12.93	.008^∗^	0.07 (−0.55∼0.69)
ΔBalance EC (mm)	11.25	−1.97	.083	0.45 (−0.18∼1.08)

Δ value was calculated as (baseline value–value at 4 wk)/baseline value × 100. Effect size was calculated with 95% CI, *P*-value at .05, and Z-value at 1.959961. CI = confidence interval, EC = sway distance on soft pad with eyes closed, EO = sway distance on soft pad with eyes open, FIQ = fibromyalgia impact questionnaire, SS = Symptom Severity Scale, VAS = Visual Analogue Scale, WPI = widespread pain index.

∗ Statistically significant (*P* < .05) difference between strengthening and stretching group.

## 4 Discussion

The European League Against Rheumatism guideline highly recommends exercise for treating fibromyalgia patients. This guideline has reported that aerobic exercise and resistance training are effective for pain and physical function improvements.^[6]^ There is evidence that exercises including strength, flexibility, range of motion and core training are beneficial to pain.^[27]^ However, the evidence of the effect and superiority of many types of exercise for fibromyalgia symptoms is insufficient.^[15–18]^

In our study, FIQ and SS were improved in the strengthening group. VAS, Borg scale, FIQ, WPI, and SS were improved in the stretching group with significant difference compared to those at baseline. Our results suggest that both types of exercise may have beneficial effects for symptoms of fibromyalgia. Although Δ value was not significantly different, mean scores of FIQ and SS were improved after exercise in each group but not in between group.

FIQ reflects beyond pain related symptoms such as physical functioning, inability to go for work, difficulty in working, and others. And SS scale represents somatic symptoms, walking unrefreshed, cognition, fatigue, and others during the last week. Many fibromyalgia patients complain of low back pain.^[28]^ And core muscle strengthening exercise is more focused on site of body that can influence balance function and lower back pain which can affect the ability of walking than a general stretching exercise program. This may have affected FIQ and SS scores in the strengthening group. And in the stretching group, exercise was focused on main areas of pain and this may have caused FIQ and SS improvement in our study.

As mentioned at outcome measures of our study, VAS was checked as average pain score during the last week and Borg scale quantifies participant's perceptions of effort during exercise. In the stretching group, exercise was focused on main areas of pain and this may have caused VAS and Borg scale improvement. Although Δ value was not significantly different, WPI was improved only in the stretching group after exercise. As mentioned in the method of exercise, general stretching exercise was focused on main areas of pain to minimize tender point location and WPI present body areas where they have pain similar to tender point. This may have affected WPI in the stretching group. Like these, each scale about fibromyalgia improved differently in each group but there was no statistical significance between groups in this study. More detailed studies are needed to supplement the limitations we mention below to investigate their effectiveness.

Besides pain related symptom in Fbromyalgia syndrome patients, postural instability and balance impairment can be accompanied. For postural stability and balance, core muscles play an important role. And other studies have reported the relationship between pain and postural control including balance.^[7]^

About balance impairment, many articles have reported that core muscle training can improve trunk muscle function and some balance test.^[29–31]^ Stimulation of muscle fibers and tactile receptors via exercise programs may cause and increase proprioception.^[12,32]^ Although Δ value was not significantly different between the 2 groups, some balance scale was improved only in the strengthening group tested on soft pad in our study. This might be due to the effect of core muscle strengthening exercise on pain related symptoms associated with somatosensory input, thus improving the balance function like in other studies.^[10,33]^

Other similar study that compared strengthening with stretching exercise in fibromyalgia with randomized controlled trial demonstrated that both exercises were efficient for fibromyalgia.^[16]^ In their study, muscle strengthening exercise focused on 12 muscle groups (gastrocnemius, tibilia anterior, quadriceps, hamstrings, gluteus, abdominalis, erector spinae, pectorals, latissimus dorsi and rhomboids, deltoids, biceps and triceps) and both strengthening and stretching exercise were effective for symptoms of fibromyalgia. However, in their study there was no significant intergroup difference.

Like that, some balance function was improved with core muscle strengthening exercise with significantly difference in our study. And other fibromyalgia related symptoms including FIQ and SS were improved in both groups while VAS, Borg scale, and WPI were improved only in the stretching exercise but neither was better with significantly different. Further study is required.

Our study presents preliminary results regarding the comparison between both exercises for fibromyalgia through a randomized controlled trial.

### 4.1 Study limitations

This study has some limitations. First, we didn’t use accelerometer used to manage physical activity outside of the intervention. In addition, we could not perform a double blinded study because the therapist must know which exercise he must do. We did not subdivide VAS into subgroups either. And stretching intervention can be more likely a light exercise than strengthening intervention. Although there was no significant difference between groups in many outcomes, most of the Δ value in this study was positive. This may have clinical relevance without having statistical significance. Second limitation of this study was the lack of long-term follow-up measures beyond 4weeks. And though we performed blinded controlled trial, there is a possibility that placebo effect or care provider contact effect could appear. Another limitation of this study is the lack of analysis about the change of muscle quality after exercise despite the change of muscle quality after exercise can be assessed by ultrasound.^[34,35]^ Further lager study and meta-analysis may be needed.

## 5 Conclusions

We concluded that the effectiveness of 4-weeks exercise program of core muscle strengthening exercise and general stretching exercise is beneficial to symptoms of Fbromyalgia syndrome independently and that core muscle strengthening is beneficial for some balance scale. Preliminary evidence suggests that both types of exercise could have positive effects on certain outcomes and we clinically recommend for exercise prescription in fibromyalgia.

## Author contributions

**Conceptualization:** Hyeng-Kyu Park, In-Sung Choi, Jae-Young Han.

**Data curation:** Hyeng-Kyu Park, Min-Kun Song, Dong-Joo Kim.

**Formal analysis:** Hyeng-Kyu Park, Min-Kun Song.

**Funding acquisition:** Hyeng-Kyu Park.

**Investigation:** Hyeng-Kyu Park, Dong-Joo Kim.

**Methodology:** Hyeng-Kyu Park, In-Sung Choi, Jae-Young Han.

**Writing – original draft:** Hyeng-Kyu Park.

**Writing – review & editing:** Hyeng-Kyu Park, Jae-Young Han.

## References

[1] Busch AJ Barber KA Overend TJ. Exercise for treating fibromyalgia syndrome. Cochrane Database Syst Rev 2007;CD003786 doi: 10.1002/14651858.CD003786.pub2. 17943797 10.1002/14651858.CD003786.pub2 PMC12832165

[2] Demir-Gocmen D Altan L Korkmaz N. Effect of supervised exercise program including balance exercises on the balance status and clinical signs in patients with fibromyalgia. Rheumatol Int 2013;33:743–50. 22580931 10.1007/s00296-012-2444-y

[3] Wolfe F Clauw DJ Fitzcharles MA. The American College of Rheumatology preliminary diagnostic criteria for fibromyalgia and measurement of symptom severity. Arthritis Care Res (Hoboken) 2010;62:600–10. 20461783 10.1002/acr.20140

[4] Maffei ME. Fibromyalgia: recent advances in diagnosis, classification, pharmacotherapy and alternative remedies. Int J Mol 2020;21:7877. 10.3390/ijms21217877 PMC7660651 33114203

[5] Hauser W Ablin J Perrot S. Management of fibromyalgia: practical guides from recent evidence-based guidelines. Pol Arch Intern Med 2017;127:47–56. 28075425 10.20452/pamw.3877

[6] Macfarlane GJ Kronisch C Dean LE. EULAR revised recommendations for the management of fibromyalgia. Ann Rheum Dis 2017;76:318–28. 27377815 10.1136/annrheumdis-2016-209724

[7] Clauw DJ. Fibromyalgia: a clinical review. JAMA 2014;311:1547–55. 24737367 10.1001/jama.2014.3266

[8] Yunus MB. Central sensitivity syndromes: a new paradigm and group nosology for fibromyalgia and overlapping conditions, and the related issue of disease versus illness. Semin Arthritis Rheum 2008;37:339–52. 18191990 10.1016/j.semarthrit.2007.09.003

[9] Oaklander AL Herzog ZD Downs HM. Objective evidence that small-fiber polyneuropathy underlies some illnesses currently labeled as fibromyalgia. Pain 2013;154:2310–6. 23748113 10.1016/j.pain.2013.06.001 PMC3845002

[10] Muto LH Sauer JF Yuan SL. Postural control and balance self-efficacy in women with fibromyalgia: are there differences? Eur J Phys Rehabil Med 2015;51:149–54. 24755776

[11] Meireles SA Antero DC Kulczycki MM. Prevalence of falls in fibromyalgia patients. Acta Ortop Bras 2014;22:163–6. 25061425 10.1590/1413-78522014220300386 PMC4108701

[12] Jones KD Horak FB Winters-Stone K. Fibromyalgia is associated with impaired balance and falls. J Clin Rheumatol 2009;15:16–21. 19125137 10.1097/RHU.0b013e318190f991 PMC2836495

[13] Goldenberg DL Burckhardt C Crofford L. Management of fibromyalgia syndrome. JAMA 2004;292:2388–95. 15547167 10.1001/jama.292.19.2388

[14] Busch AJ Schachter CL Overend TJ. Exercise for fibromyalgia: a systematic review. J Rheumatol 2008;35:1130–44. 18464301

[15] Hauser W Klose P Langhorst J. Efficacy of different types of aerobic exercise in fibromyalgia syndrome: a systematic review and meta-analysis of randomised controlled trials. Arthritis Res Ther 2010;12:R79. 20459730 10.1186/ar3002 PMC2911859

[16] Sanudo B Galiano D Carrasco L. Aerobic exercise versus combined exercise therapy in women with fibromyalgia syndrome: a randomized controlled trial. Arch Phys Med Rehabil 2010;91:1838–43. 21112423 10.1016/j.apmr.2010.09.006

[17] Jones KD Burckhardt CS Clark SR. A randomized controlled trial of muscle strengthening versus flexibility training in fibromyalgia. J Rheumatol 2002;29:1041–8. 12022321

[18] Sosa-Reina MD Nunez-Nagy S Gallego-Izquierdo T. Effectiveness of therapeutic exercise in fibromyalgia syndrome: a systematic review and meta-analysis of randomized clinical trials. Biomed Res Int 2017;2017:2356346. 29291206 10.1155/2017/2356346 PMC5632473

[19] Markovic G Sarabon N Greblo Z. Effects of feedback-based balance and core resistance training vs. pilates training on balance and muscle function in older women: a randomized-controlled trial. Arch Gerontol Geriatr 2015;61:117–23. 26036209 10.1016/j.archger.2015.05.009

[20] Garcia-Vaquero MP Moreside JM Brontons-Gil E. Trunk muscle activation during stabilization exercises with single and double leg support. J Electromyogr Kinesiol 2012;22:398–406. 22436839 10.1016/j.jelekin.2012.02.017

[21] Handa N Yamamoto H Tani T. The effect of trunk muscle exercises in patients over 40 years of age with chronic low back pain. J Orthop Sci 2000;5:210–6. 10982659 10.1007/s007760050153

[22] Richardson C Jull G Toppenberg R. Techniques for active lumbar stabilisation for spinal protection: a pilot study. Aust J Physiother 1992;38:105–12. 25025642 10.1016/S0004-9514(14)60555-9

[23] Sundstrup E Jakobsen MD Andersen CH. Swiss ball abdominal crunch with added elastic resistance is an effective alternative to training machines. Int J Sports Phys Ther 2012;7:372–80. 22893857 PMC3414069

[24] Teyhen DS Rieger JL Westrick RB. Changes in deep abdominal muscle thickness during common trunk-strengthening exercises using ultrasound imaging. J Orthop Sports Phys Ther 2008;38:596–605. 18827329 10.2519/jospt.2008.2897

[25] Gaber CE Blissmer B Deschenes MR. American College of Sports Medicine position stand. Quantity and quality of exercise for developing and maintaining cardiorespiratory, musculoskeletal, and neuromotor fitness in apparently healthy adults: guidance for prescribing exercise. Med Sci Sports Exerc 2011;43:1334–59. 21694556 10.1249/MSS.0b013e318213fefb

[26] In Heo Man-Suk Hwang Eui-Hyoung Hwang. Electroacupuncture as a complement to usual care for patients with non-acute low back pain after back surgery: a pilot randomised controlled trial. BMJ Open 2018;8:e018464. 10.1136/bmjopen-2017-018464 PMC5961607 29773696

[27] Geneen LJ Moore RA Clarke C. Physical activity and exercise for chronic pain in adults: an overview of Cochrane Reviews. Cochrane Database Syst Rev 2017;4:CD011279. 28436583 10.1002/14651858.CD011279.pub3 PMC5461882

[28] Fatemi G Fang MA Breuer P. Deconstructing chronic low back pain in the older adult – step by step evidence and expert-based recommendations for evaluation and treatment part III: fibromyalgia syndrome. Pain Med 2015;16:1709–19. 26272644 10.1111/pme.12863 PMC5066871

[29] Agmon M Belza B Nguyen HQ. A systematic review of interventions conducted in clinical or community settings to improve dual-task postural control in older adults. Clin Interv Aging 2014;9:477–92. 24741296 10.2147/CIA.S54978 PMC3970921

[30] Aladro-Gonzalvo AR Machado-Diaz M Moncada-Jimenez J. The effect of pilates exercises on body composition: a systematic review. J Bodyw Mov Ther 2012;16:109–14. 22196436 10.1016/j.jbmt.2011.06.001

[31] Amiridis IG Hatzitaki V Arabatzi F. Age-induced modifications of static postural control in humans. Neurosci Lett 2003;350:137–40. 14550913 10.1016/s0304-3940(03)00878-4

[32] Russek LN Fulk GD. Pilot study assessing balance in women with fibromyalgia syndrome. Physiother Theory Pract 2009;25:555–65. 19925263 10.3109/09593980802668050

[33] Yuan SL Berssaneti AA Marques AP. Effects of shiatsu in the management of fibromyalgia symptoms: a controlled pilot study. J Manipulative Physiol Ther 2013;36:436–43. 23830713 10.1016/j.jmpt.2013.05.019

[34] Han DS Wu WT Hsu PC. Sarcopenia is associated with increased risks of rotator cuff tendon diseases among community-dwelling elders: a cross-sectional quantitative ultrasound study. Front Med 2021;5:630009. 10.3389/fmed.2021.630009 PMC8131871 34026779

[35] Chang PH Chen YJ Chang KV. Ultrasound measurements of superficial and deep masticatory muscles in various postures: reliability and influencers. Sci Rep 2020;10:14357. 32873849 10.1038/s41598-020-71378-z PMC7463001

